# The role of system justification theory in support of the government under long-term conservative party dominance in Japan

**DOI:** 10.3389/fpsyg.2023.909022

**Published:** 2023-03-29

**Authors:** Mizuki Nakagoshi, Kazunori Inamasu

**Affiliations:** ^1^Graduate School of Sociology, Kwansei Gakuin University, Nishinomiya, Hyogo, Japan; ^2^School of Sociology, Kwansei Gakuin University, Nishinomiya, Hyogo, Japan

**Keywords:** system justification theory, political ideology, support for conservative government, general system justification, economic system justification

## Abstract

The applicability of system justification theory (SJT) in Japan, where political contexts differ from those in Western countries, was evaluated in this study. SJT explains the psychological mechanisms underlying conservatism. Japan, which has a relatively long history as a democracy among East Asian countries, has a special political context. For instance, (1) it has had almost uninterrupted conservative governance since the end of World War II; and (2) unlike Western countries, opinions on economic issues are not clearly linked to conservative attitudes. A web survey of Japanese voters (*n* = 1,428) revealed that high general system justification (GSJ) and economic system justification (ESJ) were correlated with conservatism. Further, path analysis results showed that GSJ and ESJ predicted conservative attitudes. Additional analysis suggested that the status-legitimacy hypothesis, in which lower status groups have higher system justification motives, is not supported.

## 1. Introduction

This study aimed to examine the applicability of system justification theory (SJT) to the unique case of Japan. In particular, where voters have long supported a conservative government. Further, where opinions on economic issues do not predict political attitudes, at least superficially, despite growing economic inequality.

The steps of this study are as follows. First, we provide an initial overview of SJT. Next, we discuss the significance of applying SJT to Japan. Moreover, we discuss the points to keep in mind when applying SJT to Japan, namely, the particularities of the Japanese political context and the clear differences in conservative ideology between Japan and Western countries. Through a web survey, we show that individual differences in system justification (SJ) tendencies predict conservative attitudes among Japanese voters and that SJT is applicable in Japan, an East Asian democracy.

### 1.1. System justification theory

SJT has been applied in attempts to explain why people adopt conservative attitudes that are not necessarily conducive to their self-interest, by focusing on ideological differences in cognition and needs (Jost and Banaji, 1994; Jost, 2020). SJT defines conservative ideology in terms of elements of individuals’ resistance to change and the acceptance of inequality, and studies of SJT have shown that this definition is empirically plausible when psychological concepts are used (see Jost, 2018 for a review). According to SJT, political and economic conservatism is related to people’s intrinsic, fundamental needs (Jost et al., 2003a,b,c). Hennes et al. (2012) identified three major needs that drive SJ: (i) the ontological need to recognize death and fear and to suppress associated suffering (e.g., death anxiety); (ii) the relational need to establish smooth relationships with others (e.g., the need for shared reality); and (iii) the epistemological need to recognize things as orderly and consistent (e.g., the need for cognitive closure). All of these needs are associated with conservative attitudes (Jost, 2017; Jost et al., 2018). The strength of these motives drives system justification, which in turn leads to support for a conservative regime.

Jost, an advocate of SJT, assumes that the acceptance of inequality and disparity is a central element of conservative ideology (Jost et al., 2003a,b,c; Jost, 2021). Acceptance of economic inequality, conceptualized in SJT as economic system justification (ESJ), has been reported to be associated with conservative attitudes (Jost and Thompson, 2000; Azevedo et al., 2017; Jost, 2017).

In addition, for conservatives, system justification that rationalize socioeconomic inequality satisfies these needs but also provides a high degree of well-being (Jost and Hunyady, 2002; Napier and Jost, 2008; Vargas-Salfate et al., 2018). Napier and Jost (2008) reported that in the U.S., conservative ideology predicts well-being by mediating the rationalization of economic inequality. They also showed that happiness declines as domestic economic inequality increases, and that this trend is more pronounced among liberals in Western countries. Regarding that finding, Napier and Jost (2008) pointed out that economic inequality and low well-being may be closely related in liberals because conservatives can cope with merciless realities by justifying the system, whereas liberals cannot. In other words, for conservatives, the rationalization of economic inequality is a necessary cognitive mechanism for psychological health as well, albeit to varying degrees. System justification thus fulfills some people’s fundamental needs and is a buffer that mitigates their unhappiness. SJT explains that these psychological reasons are responsible for conservatism.

System justification theory also particularly focuses on explaining the status quo motives of low-status groups. Jost et al. (2003c) suggested that low-status groups are more likely to experience ideological dissonance, and can sometimes be more strongly motivated by SJ than high-status groups. This is called the status-legitimacy hypothesis (Brandt, 2013), which posits that low-status groups show stronger resistance to social change than high-status groups, and are more likely to regard the existing social system as legitimate. Note that previous studies found that the status-legitimacy hypothesis was not supported (Brandt, 2013; Caricati, 2017). On the other hand, some argue that it would be supported under certain conditions (Sengupta et al., 2015; Vargas-Salfate et al., 2018; Li et al., 2020), and results have been inconsistent.

### 1.2. Application of SJT to the U.S. and other countries

Many examinations of the relationship between conservatism and system justification have been conducted in Western societies, especially in the U.S. (Hennes et al., 2012; Monteith and Hildebrand, 2020). For example, Azevedo et al. (2017) suggest that SJT can explain U.S. voters’ support for the Republican Party. SJT has also been applied to countries outside the U.S. For example, Caricati (2019) examined 23 major countries and reported a positive association between political conservatism and system justification (except among extreme rightists). Studies in non-U.S. contexts other than Western democratic capitalist countries have been conducted in post-communist countries such as Poland (Jaśko and Kossowska, 2013; Cichocka et al., 2015) and Hungary (Van der Toorn et al., 2010; Szabó and Lönnqvist, 2021). These studies have one thing in common: they were conducted in Western nations (see Osborne et al., 2019 for a review).

While SJT research abounds in Western countries, to the best of our knowledge, few investigations have been conducted in democratic East Asian nations. In applying SJT to East Asia, it is essential to note that the political context is very different from that of Western countries.

East Asia has a completely different setting than Western nations, where cultural values and nationality are thought to be more significant underpinnings for ideological conflict than economic issues (Dalton, 2006). As discussed below, Japan is unique in that opinions on economic issues are not linked to conservative attitudes.

Regarding SJT research in East Asia, a relatively large number of studies have been conducted in China (Tan et al., 2016; Zhang and Zhong, 2019; Du and King, 2022). However, China has an authoritarian system and must therefore be treated as a different case from democratic countries in which it is socially and institutionally acceptable to challenge the system. Few East Asian nations have long-standing, stable democracies, and little is known about how well SJT applies to these nations.

### 1.3. Reasons for focusing on Japan

The present study focuses on Japan, which has had a democratic system for more than 70 years. Japan is a unique case because, at first glance it appears to be the country to which SJT is most applicable because of the long-term stability of its conservative government. Nonetheless, it differs significantly from other countries in that economic issues are not ideological conflicts among voters on the surface. Two characteristics of Japanese politics are explained below from an SJT perspective with reference to Japan’s historical background.

First, Japan appears to be a typical case that could be explained by SJT because many Japanese voters continue to support the conservative government for a long time. Nevertheless, to the best of our knowledge, only a few studies have focused on Japanese voters.

Specifically, since World War II, Japan has been governed by a conservative camp with the Liberal Democratic Party (LDP) as its primary party (Kabashima and Takenaka, 2012). Since the LDP was formed in 1955, non-LDP governments were in power for extremely short periods of time: from 1993 to 1994 and from 2009 to 2012. In the 2019 House of Councilors regular election in Japan (which was the election held immediately after the present study was conducted), the LDP government won as usual. This is despite LDP being unfavorably affected by corruption scandals and several policy issues (Jain, 2020). The leading opposition parties–the Constitutional Democratic Party of Japan and the National Democratic Party–won a combined total of less than half the number of seats won by the LDP.

The fact that parties other than the LDP do not gain broad support among Japanese voters means that the “system” of the LDP government is extremely strong in Japan. SJT may even explain countries such as Japan better than it can explain countries with a two-party system where the “system” changes over time.

A second characteristic of Japan is that economic inequality is a familiar issue for citizens. Notwithstanding, opinions on economic issues do not predict political attitudes among Japanese voters, at least superficially (Jou and Endo, 2016).

From an international perspective, Japan’s economic disparity is severe. Regarding income redistribution, Japan has one of the highest levels of inequality among developed countries (Tachibanaki, 2006). According to data from the Organization for Economic Co-operation and Development for 2017–2018, Japan’s poverty rate is 15.7%. This is slightly lower than that of the U.S. (17.8%) but higher than that of other Western countries (Organisation for Economic Co-operation and Development, 2021).

Even though economic inequality is a familiar issue for many voters, there is no strong relationship between political attitudes and perceptions of inequality among Japanese voters. Empirical studies indicate that left–right ideology and opinions on “big government-small government” have not been closely linked in Japan for a long time (Jou and Endo, 2016). Similarly, a recent investigation showed that Japanese voters do not associate left–right ideological labels with economic issues (Miwa et al., 2021). These studies point to a persistent tendency among Japanese voters to not link ideology with economic issues.

Opinions on economic issues do not predict political attitudes because of the historical formation of ideological conflicts in Japan. In Japan, ideological conflicts formed along diplomatic and security issues under the Cold War structure. Furthermore, key ideological issues are diplomacy and security. After losing in World War II, Japan was democratized and de-militarized in the direction of the U.S. However, as the Cold War intensified, the U.S. sought to rearm Japan to position Japan as a force against communist countries. In this process, an ideological party structure was formed between the right, which favored re-armament, and the left, which opposed re-armament and affirmed post-war democracy (Kabashima and Takenaka, 2012).

Economic issues are relatively less important than diplomatic and security issues in Japan’s ideological conflict. This is also related to the LDP’s economic policy. Backed by high economic growth, the LDP has attracted voters by leveraging its abundant financial resources and redistribution of wealth to rural areas through employment. Notably, the LDP’s economic policies were not based on big government policies emphasizing social welfare but on “political clientelism,” in which the LDP is required to vote for the LDP in exchange for promises of benefits to local voters (Sunahara, 2017). Specifically, the LDP provided support to rural farmers, builders, and the self-employed in exchange for their voting for the LDP, and “redistribution” from urban to rural areas.

Furthermore, the “political clientelism” of the LDP no longer works well today and is not fulfilling its function of redistributing income. This is because of the deteriorating economic situation in Japan. Japan has experienced a pronounced economic recession since the collapse of the bubble economy in the 1990s. Additionally, the Worker Dispatch Law of 2003 has widened the gap between regular and non-regular employment in Japan, and many non-regularly employed Japanese are now suffering the brunt of corporate cost cutting (Yun, 2010; Watanabe, 2018).

The LDP’s “redistributive policies” do not target the growing number of urban voters in recent years, who are non-regular employees — voters who want to work full-time but are not employed full-time and work for low wages. In other words, for the poor in contemporary Japan, the LDP’s “redistributive policies” make little sense (Miyamoto, 2008). Despite this situation, political parties that strongly advocate for the welfare of non-regular workers have not been able to garner enough support to compete with the LDP.

We discuss the above two political characteristics of Japan. In this study, we examined whether SJT is effective in explaining the conservative attitudes of contemporary Japanese voters. Even in Japan, where opinions on economic issues have been considered not to predict political attitudes because of the historical background of ideological formation and the LDP’s “political clientelism,” it could be related to political attitudes at the level of psychological cognition, such as general system justification (GSJ) and ESJ. If SJT can be applied to Japanese voters, it would help us understand the conservatism of contemporary Japanese voters in the face of the LDP’s “political clientelism,” which is not working well and is increasingly creating inequality.

## 2. Hypotheses

This study focused on GSJ and ESJ. The reason for focusing not only on GSJ but also on ESJ is that, as we mentioned in Section 1.1, concept of conservative ideology in SJT contains the tendency to accept inequality as well as resistance to change. Therefore, ESJ, as an operative definition of acceptance of inequality, is considered an essential element in examining the applicability of SJT. If SJT is a universal theory, we should then observe a relationship between conservative attitudes and the ESJ even in Japan.

The tentative model of this study, i.e., that system justification predicts a conservative ideology, is based on Jost et al. (2017). Studies using other models include those that considered conservative ideology as preceding system justification (Jost and Thompson, 2000; Feygina et al., 2010; Moscato et al., 2021).

This study relies on the model of Jost et al. (2017) because it posits that recognizing one’s ideological stance is a complex task that requires political knowledge, which most voters do not possess. Converse (1964) found that more than 90% of American voters do not comprehend politics in abstract ideological concepts such as “conservative” or “liberal.” Luskin (1990) asserts that political sophistication, or the ability to understand politics in abstract terms and accurately identify the position of political parties and one’s opinion on policy issues, is dependent on political interest and intelligence.

We agree with the assertion that many voters have difficulty in accurately grasping the self-location of ideology. Nonetheless, we also agree with the psychological explanation that ideology exists universally among individuals (Jost, 2021). We argue that even if voters do not consciously understand “ideology” in the precise sense used by political elites, there exists system justification as a fundamental psychological mechanism. Two aspects of the study hypothesis were examined, as follows.

*H1*: High tendency for GSJ will predict support for the conservative government (the Abe Administration) mediated by conservative ideology.

*H2*: High tendency for ESJ will predict support for the conservative government (the Abe Administration) mediated by conservative ideology.

## 3. Method

### 3.1. Data collection

To test the above-described hypothesis, a web survey was conducted in June 2019. Participants were limited to Japanese citizens over the age of 18 years and recruited through crowdsourcing. A total of 1,602 responses were obtained. Among the participants, we excluded those who dropped out of the study or had missing responses. Participants who did not respond accurately to the scale of the directed questions (‘satisficers’) were also excluded from the analysis. Satisficers are participants who minimize their efforts to answer, for example, by giving a “3” to all scale items (Maniaci and Rogge, 2014). The responses of such participants may affect the survey results. To identify participants who may be satisficers, we created a one-item Directed Questions Scale: “Please answer ‘applicable’ for this item.” If this instruction is not followed, the participant is considered a satisficer.

The responses of a final total of 1,428 participants (744 women, 684 men, mean age ± standard deviation = 39.16 ± 10.24 years) were analyzed.

### 3.2. Variables

#### 3.2.1. General system justification

The GSJ scale developed by Kay and Jost (2003) was adopted, using the Japanese version provided by Murayama and Miura (2019). Participants were asked to respond to 8 items such as “In general, you find society to be fair,” and “In general, the Japanese political system operates as it should, “using a nine-point scale ranging from (1) *I do not agree at all* to (9) *I completely agree*.

#### 3.2.2. Economic system justification

Jost and Thompson’s (2000) ESJ scale was adopted. After being professionally translated from English into Japanese, the text was adjusted by authors into plain Japanese. Participants were asked to respond to 17 items, including “People can usually get what they want if they work hard” and “The disparity in wealth in society is due to the laws of nature,” using a nine-point scale ranging from (1) *I do not agree at all* to (9) *I completely agree*.

#### 3.2.3. Political ideology

Participants were asked to rate their ideological self-positioning (i.e., conservative-left self-awareness) using an 11-point scale ranging from (0) *liberal* to (10) *Conservative* for each item.

#### 3.2.4. Support for the Abe (LDP) administration

The survey asked participants to rate their support for the Abe Administration, which was in power at the time of the survey, using a four-point scale ranging from (1) *Do not support* to (4) *Support*.

#### 3.2.5. Household income

Participants were asked to respond to one item regarding their gross household income in the previous year (FY2018), using a 12-point scale ranging from (1) *Less than 2 million yen* to (12) *More than 20 million yen*. Income was log-transformed in order to make the residuals normally distributional.

#### 3.2.6. Education

Participants responded to a single item regarding their highest educational achievement, using a four-point scale ranging from (1) *Elementary/junior high school* to (4) *University/graduate school*. In the analysis, the responses were treated as continuous variables.

## 4. Results

All analyses were performed using R (version 4.1.2).

### 4.1. Scale validity

Cronbach’s alpha was calculated for each scale: GSJ was 0.82 and ESJ was 0.78, indicating a high degree of internal consistency among the scales.

### 4.2. Preliminary analysis

Simple tabulation and correlation analyses of each variable were conducted. The results are shown in Table 1.

**Table 1:** Results of the correlation analysis.

	*M*	SD	1	2	3	4	5	6	7
1 General system justification	4.08	1.14							
2 Economic system justification	5.07	0.82	0.26***						
3 Support for Abe Administration	2.47	0.88	0.52***	0.34***					
4 Conservative ideology	5.37	1.62	0.21***	0.19***	0.28***				
5 Gender (Men – 0, Women – 1)	1.62	0.50	−0.08**	−0.01	−0.10***	0.03			
6 Age	39.16	10.24	0.07**	0.15***	−0.10***	0.03	−0.07**		
7 Income	4.28	2.55	0.07*	0.17***	0.08**	0.03	0.12***	−0.09**	
8 Education	3.38	0.82	0.04	0.01	−0.07	−0.06*	−0.06*	−0.07**	0.14***
									

***p < 0.001, **p < 0.01, *p < 0.05.

Simple tabulation shows that for the main variables related to the hypothesis, such as support for the GSJ, ESJ, Abe Administration, and conservative ideology, the values reported by the participants were around the midpoint of each scale, indicating a generally neutral position. For household income, most participants reported values of 4–5 million yen, which is in-line with the median household income of 4.28 million yen in Japan (Ministry of Health, Labor and Welfare, 2018).

Next, the results of the correlation analyses between the main variables showed that, as predicted, the strength of GSJ and ESJ was correlated with conservative leanings; the stronger the GSJ, the more likely participants were to describe their ideology as conservative (*r* = 0.21, 95% CI = 0.15, 0.25) and support a conservative government (*r* = 0.52, 95% CI = 0.48, 0.55). Similarly, the stronger the ESJ, the more likely participants were to describe their ideology as conservative (*r* = 0.21, 95% CI = 0.14, 0.24) and support a conservative government (*r* = 0.34, 95% CI = 0.29, 0.39).

### 4.3. Mediation analysis

**Figure 1:**
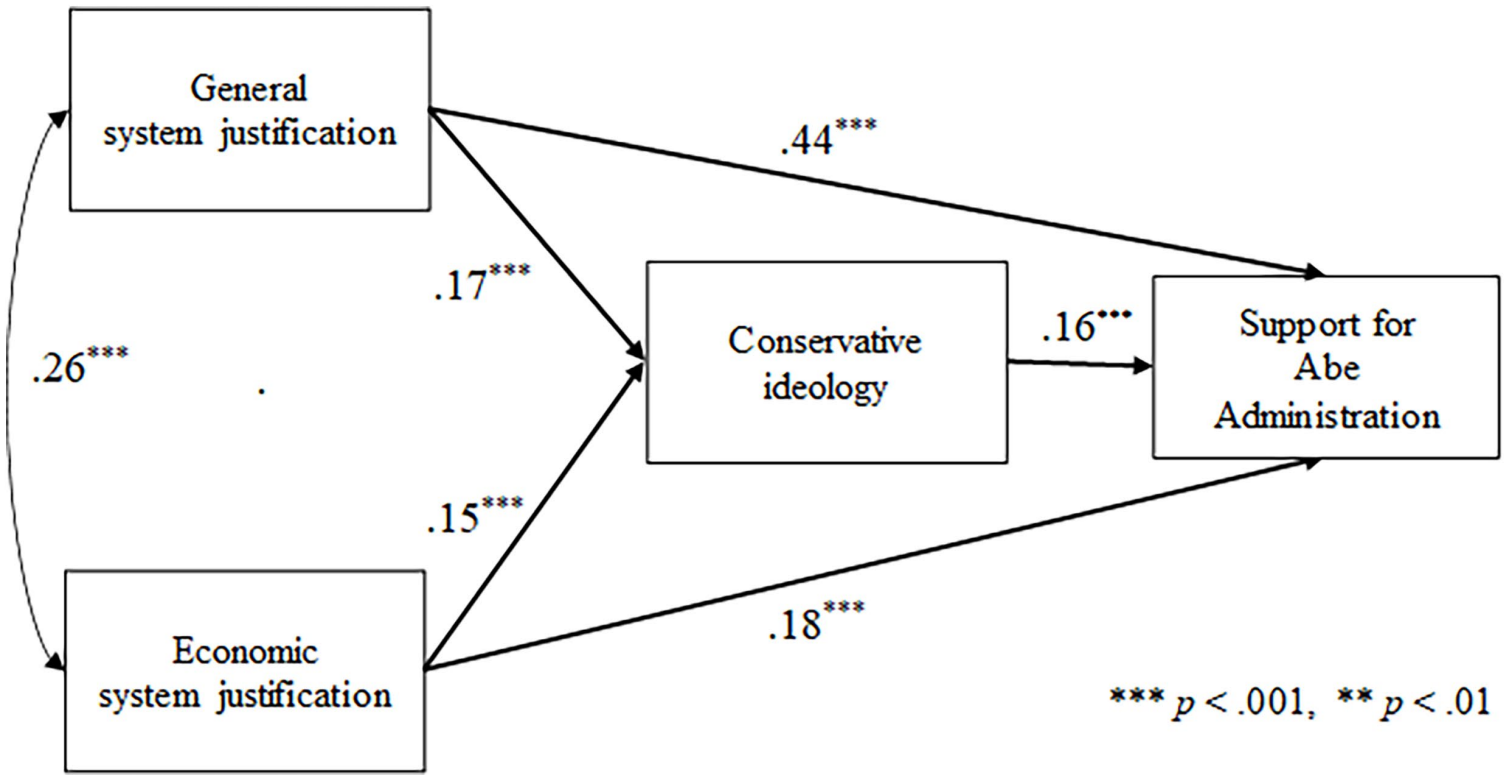
Results of the mediation analysis using standardized regression coefficients.

To test the hypothesis, mediation analysis was conducted by bootstrapping (bootstrap sample = 2000). Mediation analysis was conducted with GSJ and ESJ as the independent variables, support for the Abe Administration as the dependent variable, and conservative ideology as the mediator variable. In addition, all variables were subjected to the same path of gender, education level, household income, and age, and were included as control variables. Figure 1 shows the results of the analysis of the hypothesis model. The coefficients of the controlled variables are omitted for visibility.

#### 4.3.1. Testing hypothesis 1

The results of the analysis showed the direct effect of GSJ on support for the Abe administration was (*β* = 0.44, *p* < 0.001). There was also a statistically significant partial mediation effect (0.03, 95% CI *bs* = 0.01, 0.03, *p* < 0.001); GSJ predicted support for the Abe Administration through the mediation of the strength of conservative ideology, supporting Hypothesis 1.

#### 4.3.2. Testing hypothesis 2

The results of the analysis showed the direct effect of ESJ on support for the Abe administration was (*β* = 0.18, *p* < 0.001). There was also a statistically significant partial mediation effect (0.02, 95% CI *bs* = 0.01, 0.04, *p* < 0.001): ESJ predicted support for the Abe Administration through the mediation of the strength of conservative ideology, supporting Hypothesis 2.

#### 4.3.3. Additional analysis

We also investigated the role of demographic variables — which were treated as control variables in the hypothesis testing analysis — for system justification and support for the Abe administration. A multiple regression analysis was conducted with support for the Abe administration, the GSJ, and the ESJ as dependent variables. Gender, education, age, and income were treated as independent variables (Tables 2–4).

**Table 2:** Results of multiple regression analysis (DV: Support for the Abe administration).

DV: Support for the Abe administration			SE	*p*
Intercept	2.29	(0.05)	0.001***
Gender (0 = Men, 1 = Women)	−0.12	(0.05)	0.001***
Education	−0.04	(0.03)	0.173
Age	−0.10	(0.01)	0.001***
Income	0.11	(0.03)	0.001***
Adjusted *R*^2^ = 0.029***			

***p < 0.001.

**Table 3:** Results of multiple regression analysis (DV: GSJ).

DV: General system justification		SE	*p*
Intercept	4.08	(0.03)	0.001***
Gender (0 = Men, 1 = Women)	−0.08	(0.06)	0.003**
Education	0.03	(0.04)	0.321
Age	0.07	(0.01)	0.006**
Income	0.08	(0.05)	0.002**
Adjusted *R*^2^ = 0.015***			

***p < 0.001, **p < 0.01.

**Table 4:** Results of multiple regression analysis (DV: ESJ).

DV: Economic system justification		SE	*p*
Intercept	4.83	(0.05)	0.001***
Gender (0 = Men, 1 = Women)	−0.03	(0.04)	0.198
Education	−0.02	(0.03)	0.410
Age	−0.14	(0.01)	0.001***
Income	0.16	(0.03)	0.001***
Adjusted *R*^2^ = 0.044***			

***p < 0.001.

First, gender negatively predicted support for the Abe administration and the GSJ/ESJ. That is, male participants tended to support the Abe administration and have higher GSJ/ESJ than female participants. Education level positively predicted GSJ, while it negatively predicted support for the Abe administration and ESJ. However, education level did not have a significant effect in either analysis. Regarding age, lower age positively predicted support for the Abe administration and ESJ, while it negatively predicted GSJ. Still, the coefficients were small, and thus it may not be possible to conclude that there is a substantial effect of age. Finally, looking at income, higher income consistently positively predicted support for the Abe administration as well as GSJ/ESJ. The participants with higher incomes tended to be more conservative than those with lower incomes.

## 5. Discussion

This study tested the generalizability of SJT in a political-psychological context, using the case of Japan. The results of the web survey confirmed the process by which the GSJ and ESJ predicted support among Japanese voters for a conservative government *via* conservative ideology. The results have two important implications.

First, SJT may shed light on the current situation in Japan, where liberal parties face difficulty gaining widespread support from voters. Conservative governments remain in power for extended periods, despite poverty becoming a pressing social issue. It is hypothesized that individuals are strongly inclined to legitimize systems that significantly impact their lives, such as the government, on which they are heavily dependent (Van Der Toorn et al., 2011; Friesen et al., 2019). Although this is a matter of conjecture, a prolonged conservative regime, such as the Japanese one, may have increased people’s dependence on the system over time. Consequently, their perception of the legitimacy of the political system may have strengthened. This study suggests that the seemingly bizarre maintenance of Japan’s political system may be sustained by voters’ psychological mechanisms, which may make the rise of liberal political parties more challenging.

Second, the differences in political attitudes arising from SJ mechanisms may be a universal phenomenon. The present finding that the concept of SJT (especially ESJ) is applicable in the unique Japanese context suggests that SJ tendencies can exist as an individual difference factor underlying political attitudes beyond the political context.

However, the strength of the degree to which SJ predicts political attitudes may be context-dependent. The mediation effect of SJ in predicting support for conservative governments through ideology was remarkably weak. This may reflect the peculiarities of the electorate. Unlike the U.S., Japan has a multiparty system. Additionally, unlike voters in the U.S., where ideological conflicts between political parties are apparent and voters are likely to clearly express their own ideology, Japanese voters who have difficulty understanding conflict between political parties from an ideological perspective may also have ambiguous ideological self-placement. Given that the SJ scale can measure the underlying political orientation of voters in many countries, it may be a more useful predictor of voters’ political opinions than ideological self-placement.

Moreover, an additional analysis suggests that the status-legitimacy hypothesis is not fully supported, at least in Japan. Based on this hypothesis, it would be expected that lower-income voters would tend to be more conservative, but the results actually show the opposite. Many previous studies also do not support the status-legitimacy hypothesis. Simultaneously, the present study suggest that the status-legitimacy hypothesis is not unconditionally affirmed.

As a counterargument to the status-legitimacy hypothesis, Jost (2018) emphasized the need to consider that system justification is a composite of various psychological motives, and support in low-status groups may be reduced by other psychological motives, such as in-group favoritism. In light of this view, it is unlikely that the situation assumed by the status-legitimacy hypothesis will always hold.

### 5.1. Study limitations

There are several study limitations to consider. A single item was used to ask the participants about their self-awareness ideology. This method has frequently been used in major social surveys, such as the World Values Survey, and it was necessary here to characterize the work as an ideological study from a psychological perspective. However, there is still some controversy regarding whether single-item measurement is appropriate. For example, it is undeniable that individuals who cannot accurately grasp their own ideology may simply select the midpoint. In addition, it is known that the ideology of the Japanese is not as extreme as that of U.S. citizens, and thus many of the Japanese participants answered items by selecting the midpoint. The present results must be interpreted in light of this. To overcome this problem, it is necessary to incorporate a method to measure ideology as a bundle of political issues by measuring attitudes on several political issues.

There is also the question of whether the model is appropriate. In this study, we used a model in which SJ predicts ideology. However, as noted earlier, since there are also models in previous studies in which ideology predicts SJ, the present study supplemented the analysis with a different model (see Supplementary material). The lack of agreement within SJT studies on a model of the relationship between ideology and SJ tendency is concerning; it may be necessary to establish a more generalizable and sharable theoretical model.

## Data availability statement

The raw data supporting the conclusions of this article will be made available by the authors, without undue reservation.

## Ethics statement

The studies involving human participants were reviewed and approved by Kwansei Gakuin University Division for Research Development and Outreach. Written informed consent for participation was not required for this study in accordance with the national legislation and the institutional requirements.

## Author contributions

MN and KI contributed to the conception and design of the study. MN organized analysis of the articles and wrote the manuscript. All authors contributed to the manuscript revision, read it, and approved the submitted version.

## Funding

This work was supported by the Japan Society for the Promotion of Science #22J12212 to MN and #22H00812 to KI.

## Conflict of interest

The authors declare that the research was conducted in the absence of any commercial or financial relationships that could be construed as a potential conflict of interest.

## Publisher’s note

All claims expressed in this article are solely those of the authors and do not necessarily represent those of their affiliated organizations, or those of the publisher, the editors and the reviewers. Any product that may be evaluated in this article, or claim that may be made by its manufacturer, is not guaranteed or endorsed by the publisher.

## Supplementary material

The Supplementary material for this article can be found online at: https://www.frontiersin.org/articles/10.3389/fpsyg.2023.909022/full#supplementary-material
