# Supplementary material for: The role of system justification theory in support of the government under long-term conservative party dominance in Japan
Source: Front Psychol. 2023 Mar 29;14:909022. doi: 10.3389/fpsyg.2023.909022 (PMC10095829; doi:10.3389/fpsyg.2023.909022)
Supplement: Supplementary file 1 [file Presentation_1.pdf]

Supplementary Materials

Analysis of a model of ideology predicting system justification

Some analyses have identified conservative ideology as a precursor to system justification (Feygina, Jost, & Goldsmith, 2010; Jost & Thompson, 2000; Moscato, Caricati, & Bonetti, 2021). We also tested a model in which ideology predicts support for a conservative administration through general system justification (GSJ) and economic system justification (ESJ).

The mediation analysis was conducted by bootstrapping (bootstrap sample = 2,000) with ideology as the independent variable, support for the Abe administration as the dependent variable, and system justification as the mediator variable (Fig. 2). All variables were subjected to the same path of gender, education level, household income, and age, and were included as control variables.

FIGURE2

Results of the mediation analysis of a model for ideology predicting SJ.

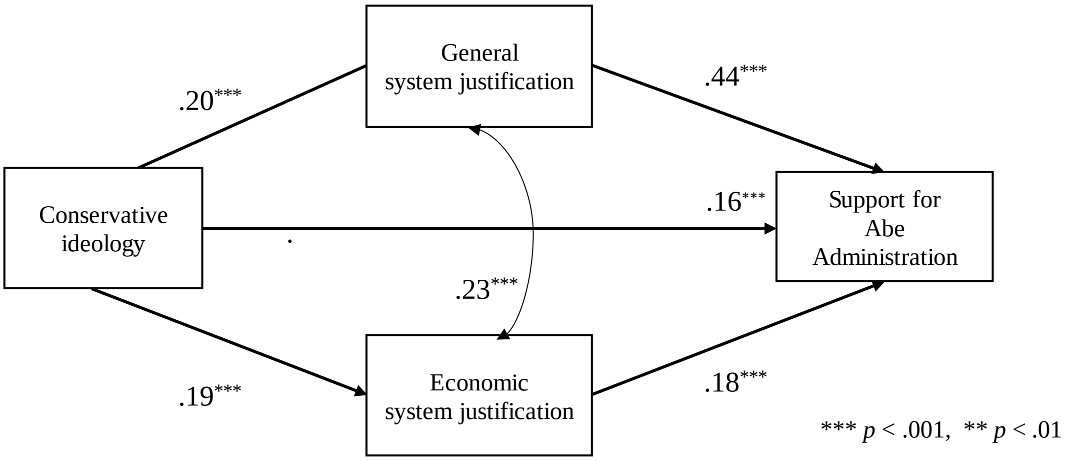

The results of the analysis showed the direct effect of conservative ideology on support for the Abe administration was (β = .16, $p < .001$). There was a statistically significant partial mediation effect (.05, 95% CI *bs* = .04, .07, $p < .001$); conservative ideology predicted support for the Abe administration through the mediation of the strength of GSJ. There was also a statistically significant partial mediation effect (.02, 95% CI *bs* =.01, .03, $p < .001$): conservative ideology predicted support for the Abe administration through the mediation of the strength of ESJ.

Both the model used in the analysis and that used in the Appendix are saturated models (equivalence models), and all paths are significant. For this reason, it is difficult to compare models with reference to goodness of fit, and we can only judge which model is more appropriate based on the content validity. We support the model presented in this article in which system justification predicts ideology, although it is impossible to say from this study alone whether our model is better for other SJT studies.

Sample representativeness

This was a web survey of the Japanese registered with crowdsourcing service. Thus, possibile bias exists in the sample. Therefore, we checked for sample bias regarding age, gender, education, and income. Specifically, we compared data provided by Japanese administrative agencies with those from this survey.

The analysis showed that the participants in this study were slightly younger and more educated than a representative sample in Japan. Further, they were in the median income brackect. Future research should be based on random sampling from Japanese population to avoid our study bias and confirm the robustness of our findings.

Data

Analysis of age, gender, and education was taken from the 2020 Census data provided by the Statistics Bureau of Japan (Statistics Bureau of Japan, 2020). This survey is a complete enumeration of all persons living in Japan.[1] However, for this analysis, we only used data from those with Japanese nationality.

For income analysis, we used data from the 2019 comprehensive survey of living conditions (Ministry of Health, Labour and Welfare, 2019). [2]This is because the Japanese National Census does not have an item on income. Notably, although the Comprehensive Survey of Living Conditions is not a complete population survey like the census, it is highly representative data.

[1] The collection rate for the 2020 Census was 67.7%.

[2] The collection rate for the 2019 National Survey of Living Standards was 72.9%.

Table5

Table of the mean age in our survey and in the national census (standard deviations are shown in parentheses)

	Data			
	Original Survey		National Census	
Age	39.16	(10.24)	47.08	(24.23)

Table6

Crosstabulation table by gender (the category ratios in the column are shown in parentheses)

		Data			
		Original Survey		National Census	
Gender	Men	684	(47.90%)	59,075,539	(48.61%)
	Women	744	(52.10%)	62,465,616	(51.39%)
Column Total		1,428	(100%)	121,541,155	(100%)

Table7

Crosstabulation table by education level (the category ratios in the column are shown in parentheses)

	Data			
	Original Survey		National Census	
Elementary/Junior high school	15	(1.05%)	12,068,422	(14.08%)
Senior high school, etc.	263	(18.42%)	37,845,056	(44.16%)
Junior college/Technical college	315	(22.06%)	13,890,514	(16.21%)
University/graduate school	835	(58.47%)	21,899,942	(25.55%)
Column Total	1,428	(100%)	85,703,934	(100%)

Table8.

Crosstabulation table by household income. Numbers in parentheses indicate the ratio of the category in the column.

		Data			
		Original Survey		Comprehensive Survey of Living Conditions	
Household Income	Less than 200 (Unit:10,000 yen)	195	(13.66%)	1,902	(19.02%)
	200 - 300	208	(14.57%)	1,355	(13.55%)
	300 - 400	236	(16.53%)	1,285	(12.85%)
	400 - 500	224	(15.69%)	1,046	(10.46%)
	500 - 600	170	(11.90%)	868	(8.68%)
	600 - 700	98	(6.86%)	807	(8.07%)
	700 - 800	97	(6.79%)	617	(6.17%)
	800 - 1000	113	(7.91%)	893	(8.93%)
	1000 - 1200	36	(2.52%)	507	(5.07%)
	1200 - 2000	42	(2.94%)	593	(5.93%)
	2000 or more	9	(0.63%)	125	(1.25%)
Column Total		1,428	(100%)	9,998	(100%)

References

Ministry of Health, Labor and Welfare (2019). Comprehensive Survey of Living Conditions. Retrieved Dec 7, 2022 from https://www.mhlw.go.jp/english/database/db-hss/cslc-tables.html

Statistics Bureau of Japan (2020). 2020 Summary of the results and statistical tables. Retrieved Dec 7, 2022 from https://www.stat.go.jp/english/data/kokusei/2020/summary.html
